# Does Eagerness for Physical Activity Matter? The Association Between Eagerness and Physical Activity Among Adolescents

**DOI:** 10.3389/fpubh.2019.00088

**Published:** 2019-04-18

**Authors:** Hilde Kristin Mikalsen, Pål Lagestad, Marte Bentzen, Reidar Säfvenbom

**Affiliations:** ^1^Department of Physical Education and Sport Science, Faculty of Education and Arts, North University, Levanger, Norway; ^2^Department of Physical Education and Pedagogics, Norwegian School of Sport Science, Oslo, Norway

**Keywords:** physical activity, eagerness, athletic competence, parental support, health

## Abstract

This study investigated the association between perceived parental support and eagerness for physical activity (EPA) among adolescents, and the association between EPA and physical activity. We further examined whether perceived athletic competence (PAC) mediates a presumed association between EPA and physical activity level, when controlling for gender. This study included 320 adolescents (aged 12–13) from 18 schools in two medium-sized Norwegian municipalities. Questionnaires and accelerometers were assessed during spring 2017. Structural equation modeling was applied to examine the associations. Standard regression coefficients are presented. Data demonstrated that perceived parental support was positively associated with EPA (β = 0.52), and eagerness was positively associated with PAC (β = 0.52). Both EPA (β = 0.20) and PAC (β = 0.24) were also positively associated with moderate to vigorous physical activity (MVPA). When mediating the relationship between EPA and MVPA, through PAC, the effect on MVPA increased (from β = 0.20 to β = 0.32). Analysis revealed that 87% of the adolescents fulfilled the national health recommendations for physical activity. This study highlights the relevance of EPA and PAC in studies of physical activity among adolescents, and the impact of perceived parental support for adolescents' EPA and physical activity level.

## Introduction

Physical inactivity has proven to be a significant health-related challenge (1–4), and has even been described as a global pandemic by several researchers (5, 6). Research that aims to understand involvement in physical activity, and reduction in physical activity during adolescence in particular (7–9), indicates that involvement in physical activity is an expression of complex and dynamic factors in, and between, individuals and the environment (10–12). According to research showing that physical activity habits in early childhood and adolescence appear to influence physical activity habits later in life (13–15), it is crucial to understand the relational interplay between significant activity promotive factors in this cohort. Individual eagerness for physical activity (16), received parental support (17), and perceived athletic competence (18) have been demonstrated to contribute to human functioning and health. The purpose of this study is therefore to elucidate the relationship between these three variables and physical activity level in adolescents. In order to increase our knowledge about which factors promote activity for whom, and in which phase of life (19–21), a better understanding of the relationships between these variables may contribute to identify presumed important variables in the relationship between internal developmental resources of adolescents (22), and external developmental resources in their local community, such as sports clubs and educational institutions.

The concept of “eagerness for physical activity” (EPA), represent a way of identifying a drive for physical activity behavior that contrasts instrumentally or rationally driven behaviors (16). The concept is theoretically anchored in lived experiences (16, 23), in which lived experiences are understood to constitute the individual's reference and assessment base when encountering new experiences. EPA reflects a positive mental state, characterized by delight, passion and deeply felt longing, or desire for something that does one good (16). Desire is assessed by Jensen (24) as a key concept in understanding people's drive for learning and development, and according to Higgins et al. (25), this mental state of eagerness is associated with the promotion of positive behavior, rather than prevention of negative behavior. Eagerness, as a regulatory orientation, is thus directed toward behaviors that are assessed to be of personal relevance, or in it selves meaningful. Accordingly, the concept of eagerness for physical activity describes the motivation for a behavior that is satisfying in its own right. Furthermore, the psychological qualities inherent in EPA (i.e., hope and positive intention to maintain physical activity in the future) are presumed to possess significant potential to predict sustainable involvement and participation in physical activity. Säfvenbom et al. (16) revealed that EPA manifests itself in higher levels of VO_2_max, and that eagerness for physical activity has predicative validity above and beyond self-determination motivation.

From a relational developmental system perspective, which Lerner and Overton (26) call for in studies of youths' developmental processes, parents play a significant role in influencing their offspring's knowledge, competences, values, and attitudes (27). A review-study (17) supports this assertion, finding parental support to be the most important socio-contextual variable relating to adolescents' motivation for physical activity. Another review study by Jaeschke et al. (28) finds that parents influence the activity behavior of their offspring by reinforcing psychosocial qualities, such as increased self-efficacy and perceived athletic competence. Parents' role in influencing their offspring is according to Fredricks and Eccles (29) classified into different mechanisms, such as providers and interpreters of experiences, and as role models. However, parental influence on activity behavior, as it is perceived by their teenage offspring, is assessed to be an important aspect (30, 31). Even though parental influence diminishes with increasing age (32), a study by Norton et al. (33) reports that parental influence on children's behavior extends beyond adolescence. Given the biological and environmental changes that occur during this period of life, further investigation is needed to illuminate the impact of the parental–adolescent relationship, on the adolescents' physical activity behavior.

Perceived athletic competence (PAC) is described as important mental capital in the context of development and learning (20, 34). According to Harter (18), PAC is an age-dependent cognitive and social construction which influences future actions, based on a basic human urge to protect and enhance one's self-perception (18). PAC is acknowledged as a highly important correlate of physical activity during adolescence (32, 35–37). Moreover, according to Weiss and Phillips (17), PAC is the most significant individual variable to understand the decline in young people's physical activity level, referring to “beliefs, judgments, and feelings about one's physical abilities and competencies in general or in a particular domain.” However, movement contexts differ (38), and prior studies indicate that PAC as a predictor for participation in competitive youth sports differs from PAC as a predictor for participation in self-organized activities (39, 40), and physical education in school (41).

**Figure 1:**
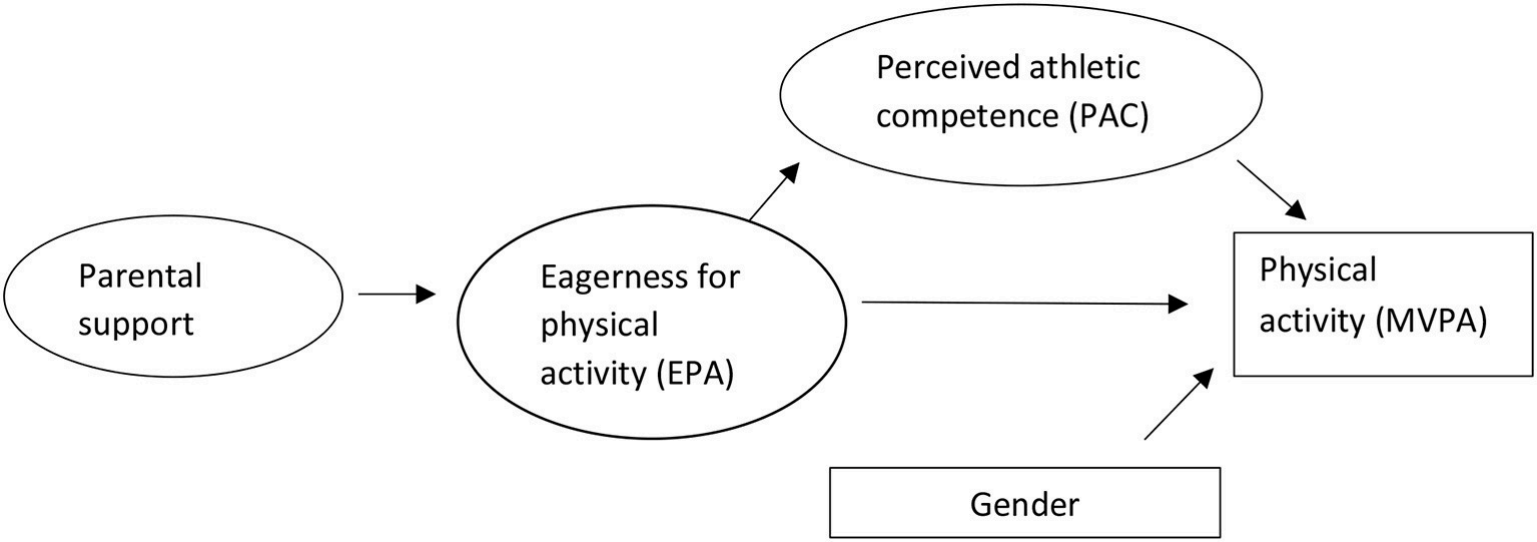
Proposed model of the study variables.

As introduced, EPA and PAC are presumed to be relevant predictors of adolescents' physical activity levels. Until now, no studies have examined the association between EPA and physical activity levels in 12–13-years-old, or between EPA and PAC. It may also be worthwhile to investigate the impact of perceived parental support on this cohort's EPA; how do humans, in the transition between childhood and adolescence, perceive and adopt their parents physical activity- related attitudes and behaviors in their own values, appreciations and intentions to be a physically active person? According to extant literature that has identified PAC to be a correlate of physical activity, PAC is suggested as a variable with the potential to mediate the relationship between 12- and 13-years-old adolescents' EPA and their moderate to vigorous physical activity level (MVPA). The aim of this study is therefore 3-fold:

- Examine the physical activity level among adolescents aged 12–13-years-old.
- Investigate the relationships between the directs paths of the proposed SEM-model in Figure 1.
- Investigate whether PAC is a mediator in the relationship between EPA and MVPA, when controlling for gender.

The correlates of the study, as explained in the introduction, are presented in Figure 1.

## Materials and Methods

### Participants and Procedures

The data material in this cross-sectional study comprises accelerometer measures and questionnaire surveys from 320 participants, 161 girls and 159 boys, aged 12–13 years. All the data material was collected during spring, 2017. The sample (77% of the cohort) comes from 18 schools in two medium-sized Norwegian municipalities (~15–22,000 inhabitants). Sixty three percentages come from schools close to a city, while 117 pupils (37%) attended more rural schools. The sample is considered random according to the available population (42, 43). The study has been approved by the Norwegian Social Science Data Service (NSD), and the parents and youths have given their written informed consent to participate.

### Measures

In accordance with the recommendations physical activity levels for children and young persons from the Norwegian Directorate of Health (44), and previous studies of physical activity (1, 8), physical activity level is presented as moderate to vigorous physical activity (MVPA), measured using an accelerometer (Actigraph GT1M). Operationalizing physical activity level into MVPA makes data comparable to the relevant previous studies (8, 45). The youths were instructed to wear the accelerometer on the right hip for 7 days consecutively at all times, except during water activities or while sleeping. According to the test protocol, a daily wear-time of 8 hours for a minimum of 2 days was set as a criterion for a valid measurement (8). The activity level was registered as counts per minute (cpm), and average cpm for valid days (≥2) was applied. Cut-off for MVPA was set in line with a Norwegian population study (8), with intervals of 2,000 counts or more. Periods with zero registrations for more than 20 min and the period between 12:00 and 6:00 am were not included.

The questionnaire has been designed with previously validated scales for the different variables. The questions in the scales have closed response alternatives designed with four, five or seven Likert scale alternatives (43), with neutral middles in the five and seven options scales.

*Eagerness for physical activity* (EPA) was measured using the “Eagerness for Physical Activity Scale” (EPAS) (16). This scale has nine items, aimed at measuring affective and cognitive aspects, such as the person's desire to be physically active, the person's delight, meaning- and identity-making in/through physical activity, as well as behavioral aspects, such as the person's hopes and intentions to maintain physical activity in the future. The items are designed as statements, such as “I always look forward to training or being physically active,” and seven response alternatives, where 1 is “Disagree completely,” and 7 is “Agree completely.” Since the validation of EPAS in 2016 (16), EPAS has been applied on two different samples of Norwegian youth in secondary schools (46) and upper secondary schools (47). These studies confirmed high internal consistency with a Cronbach's alpha above 0.9, thus indicating a reliable measurement model.

*Perceived athletic competence* (PAC) was measured by means of five items, from Harter's Self-Perception Profile for Adolescents (18). These five items measuring perceived athletic competence, is Wichstrøms (48) edited and translated Norwegian version of one of eight subscales in Harter's Self-Perception Profile for Adolescents. The items were designed as statements, such as “I'm good at all sorts of sports,” and four alternative responses, where 1 is “Agree very little” and 4 is “Agree very much.” Harter's Self-Perception Profile (18) instrument has been previously used in several studies of children and adolescent's PAC (49–51).

*Parental support (PS)* was studied as an influencing variable related to eagerness. PS was measured by means of six items modified from a prior study measuring parental support for movement activities (41). These were designed as statements, such as “Dad has always supported my physical activity,” and seven alternative responses, where 1 is “Disagree completely,” and 7 is “Agree completely.”

*Gender* is applied as a control variable for MVPA, as previous studies report conflicting evidence for gender as a predictor of the physical activity level of young people (8, 52, 53).

### Data Analysis

The collected data were screened according to “missing” and “normality” using SPSS [version 24, (54)]. A maximum of 2.5% of the data was missing on a single item for each variable used. Little's Missing Completely At Random (MCAR) test was utilized to determine whether there were patterns in the missing data. However, the results indicated that the data were completely missing at random, $\chi_{\left(374\right)}^{2}$ = 319.42, *p* = 0.981. In addition, the data were considered to be normally distributed on the single-item level in terms of skewness (range −1.84–0.38) and kurtosis (range −0.77–3.11) (55, 56). M*plus* (M*plus* version 8.0) (57) was used when evaluating the factor structure for the instruments according to Confirmatory Factor Analysis (CFA), in which the first indicator approach was employed to set the matrix with maximum likelihood robust (MLR) estimation (58). After looking for acceptable model fit for the latent variables (56, 58), internal consistency for the scales for the latent variables was also determined by checking alpha in SPSS [Chronbach's alpha ranged from 0.75 in PAC to 0.93 in EPA (59)]. Descriptive statistics on the study variables were computed and presented as means and standard deviations. Thereafter, a bivariate correlation was conducted to explore relations between the latent variables, and Student's *t*-tests for two independent samples were performed to elucidate gender differences in the study variables. The full structural model was tested using MLR estimation in M*plus*. In addition, the bootstrapping methodology for mediations with 10,000 bootstraps was performed to search for additional indirect effects in the model (60). These combinations of fit indices were utilized to evaluate acceptable model fit for all analyses conducted in M*plus* (58); Comparative Fit Index (CFI) ≥ 0.90, Tucker–Lewis Index (TLI) ≥ 0.90, Standardized Root Mean Square Residual (SRMR) ≤ 0.08, and Root Mean Square Error of Approximation (RMSEA) ≤ 0.06.

## Results

As shown in Table 1, the preliminary CFA determining the factor structure of the latent variables of parental support and eagerness demonstrated a very good fit to the data (see Table 1). However, the CFA for athletic competence indicated that two of the items had parameter estimates below 0.3 (item 4 = 0.30 and item 5 = 0.27). Kline (56) recommends that items <0.5 should not be kept, and thus these items were deleted from this latent variable. By reducing this scale to three items, the goodness-of-fit evaluation does not apply to variables with only three items, as this type of solution is described as “just-identified” (58). Thus, variables with three items can still be evaluated in terms of interpretability and strength on their parameter estimates. For the three item constructs of athletic competence, the standardized parameter estimates were 0.79, 0.77, and 0.60, respectively, thus explaining 60–79% of the variance in the latent construct.

**Table 1:** Results from the confirmatory factor analysis.

**Variable**	****χ^2^** (df)**	**CFI**	**TLI**	**RMSEA (90% CI)**	**SRMR**
Parental support	5.72 (4)^*^	0.99	0.99	0.04 (0.00–0.10)	0.02
Eagerness	57.56 (25)^*^	0.97	0.96	0.06 (0.04–0.09)	0.03
Athletic competence	0 (0)^*^	1.00	1.00	0.00 (0.00–0.00)	0.00

* *<0.05; χ^2^, chi square; df, degrees of freedom; CFI, comparative fit index; TLI, Tucker-Lewis index; RMSEA, root mean square error of approximation and 90% confidence interval; SRMR, standardized root mean square residual*.

87% of the study participants met the national recommendations for physical activity, although boys exhibited a significantly higher physical activity level (M = 94.43, SD = 30.71) compared to girls (M = 86.67, SD = 24.51): [*t*_(300)_ = −2.43, *p* = 0.016] (Table 2). As can be seen in Table 2, Student's *t*-tests showed a significant difference [*t*_(300)_ = −2.43, *p* = 0.016] between boys' and girls' physical activity level. In addition, mean-estimations of eagerness revealed that both boys and girls reported an average score of 82% of the maximum score level for eagerness for physical activity (M = 5.72, SD = 1.19, max score = 7). For perceived athletic competence and parental support, the mean score was also >50% of the maximum score. The analysis revealed no significant gender differences in the three independent variables.

**Table 2:** Student's *t*-tests for two independent samples on differences of gender on the study variables.

	**Girls**			**Boys**						
	**Mean**	**SD**	***n***	**Mean**	**SD**	***n***	***t***	**DF**	**P**	**ES**
MVPA	86.67	24.51	155	94.43	30.71	147	−2.43	300	0.016	0.279
EPA	5.82	1.09	156	5.62	1.28	158	1.481	312	0.14	0.168
PAC	2.44	0.56	157	2.56	0.71	155	−1.63	310	0.104	0.121
PS	5.75	1.23	157	5.54	1.18	151	1.54	306	0.125	0.174

*M, mean; SD, standard deviation; DF, degrees of freedom; ES, effect size (Cohen's d); MVPA, moderate-to-vigorous physical activity level; EPA, eagerness for physical activity; PAC, perception of athletic competence; PS, parental support*.

As can be seen in Table 3, all variables were positively correlated with each other, as presumed according to the theoretical model (Figure 1). Using interpretation of correlations, according to Hopkins et al. (61), the analysis identified moderate correlations between PAC and eagerness (*r* = 0.52^***^), and between parental support and eagerness (*r* = 0.52^***^).

**Table 3:** Estimated correlation matrix for the latent variables.

**Variable**	**M**	**SD**	**α**	**1**	**2**	**3**
1. Physical activity	90.45	27.93	–	–		
2. Athletic competence	2.50	0.64	0.75	0.34^***^	–	
3. Eagerness	5.72	1.19	0.93	0.32^***^	0.52^***^	–
4. Parental support	5.65	1.21	0.86	0.17^***^	0.26^***^	0.52^***^

N = 320;

*** *p < 0.001; SPSS 24 was used to calculate the means and standard deviations reported, as the means of latent variables are zero in cross-sectional studies*.

**Figure 2:**
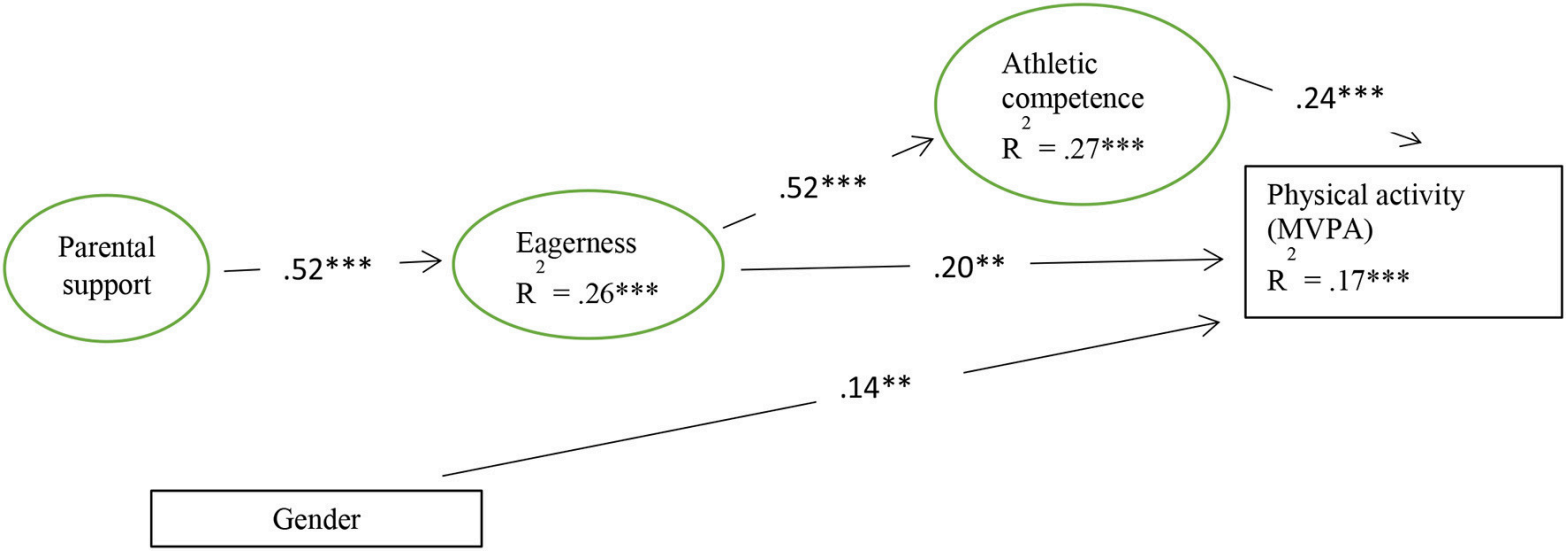
Standardized values for the structural model. Only statistically significant paths are shown. **p < 0.01, ***p < 0.001.

Five paths were specified in M*plus* to test the hypothesized model: three paths to the dependent variable MVPA from the two independent variables, PAC and eagerness, and the control variable gender, one path to PAC from eagerness, and one path to eagerness from parental support. This model yields a good fit to the data: $\chi_{\left(160\right)}^{2}$ = 283.17, *p* < 0.001, CFI = 0.96, TLI = 0.95, RMSEA = 0.05 (90% CI = 0.04–0.06), SRMR = 0.05. As can be seen in Figure 2, the explained variance (R^2^) for the variables in the model were: 17% for physical activity, 26% for eagerness, and 27% for athletic competence.

Figure 2 also shows that parental support was positively associated with eagerness (ß = 0.52), and eagerness was positively associated with PAC (ß = 0.52). Moreover, both eagerness (ß = 0.20) and athletic competence (ß = 0.24) were positively associated with MVPA. In accordance with the findings of the *t*-tests, gender also yielded a positive association with MVPA (ß = 0.14), although in a weaker manner compared to the other significant relations in the model.

Furthermore, an additional indirect effect from eagerness through PAC on MVPA was examined by adding this indirect effect to the structural model, as recommended by Hayes (62). This analysis also yielded a good fit to the data for the structural regression model using 95% bias-corrected bootstrap CI derived from 10,000 resamples: $\chi_{\left(160\right)}^{2}$ = 339.44, *p* < 0.001, CFI = 0.95, TLI = 0.94, RMSEA = 0.06 (90% CI = 0.05–0.07), SRMR = 0.05. The results from this test indicated that the total standardized association from eagerness to MVPA increased (ß = 0.32, *p* < 0.001) due to an additional indirect effect through PAC on MVPA (ß = 0.12, *p* < 0.01). The direct path from eagerness to PAC remained unchanged in this analysis (ß = 0.20, *p* < 0.01).

As the current study is a cross-sectional study [according to Atkin et al. (11), cross-sectional studies do not allow for causal interference to be made], an alternative model yielding alternative theoretical implications was tested to control for confirmation bias (63). The alternative model tested was the same except for the change of places between PAC and eagerness in the model. The results for this model did not yield an acceptable fit to the data: $\chi_{\left(160\right)}^{2}$ = 336.73, *p* < 0.001, CFI = 0.94, TLI = 0.93, RMSEA = 0.06 (90% CI = 0.05–0.07), SRMR = 0.13.

## Discussion

The findings show that 87% of the youths fulfilled the national health recommendations for physical activity. Moreover, our data demonstrated that eagerness, PAC, and gender were positively associated with MVPA. Parental support was also positively associated with eagerness, and eagerness was positively associated with PAC. When mediating the relationship between eagerness and MVPA, through PAC, the effect on MVPA increased (from β = 0.20 to β = 0.32), although the path from eagerness to MVPA remained unchanged. This finding will be discussed further below.

The objective measurements of the physical activity level of the youths, showing that 87% satisfied the national recommendations for 60 min of MVPA per day (44), are substantially higher than what could be anticipated when compared to previous studies of adolescents' physical activity level (1, 8). In Kolle et al.'s study, 86/70% of the 9 years-old boys/girls, and 58/43% of the 15 years-old boys/girls fulfilled the national recommendations (44). It should, however, be noted that this study differs from a previous Norwegian study (8) in the age of the cohort (12–13 vs. 9 and 15 years old). One possible explanation could therefore be related to an increase of time for physical activity in the 5th−7th grade in primary school (64). Recent debates among educators and researchers (65) concerning the health benefits of physical activity can be considered another. This sample's conditions for activities of daily living are assumed to be no different from any other samples belonging to similar municipalities.

Another issue to address is the possibility of presenting a magnified picture of the average physical activity level throughout the year, as this data material was collected only during the spring. Previous studies (8, 66, 67) have revealed seasonal variations in physical activity level, finding that 6–10 years-old children were more physically active during spring and summer. These differences were however not present among Norwegian and Danish youths from 14 to 16 years (8, 66). Accordingly, seasonal variation can be a possible bias in this sample, but we find it reasonable to believe that the physical activity level of these 12–13 years old youths is more likely to be representative for the whole year, than if they had been younger.

A suggest that the 12–13-years-old youths in the study, in general, assess their personal relationship to physical activity as positive. This indicates that physical activity is generally experienced as exciting and personally relevant or meaningful in the present, and that their experiences also nurtures their hopes and intentions for long-term physical activity. The SEM analysis (Figure 2) finds a positive association between EPA and MVPA. This was expressed by a one standard deviation higher score of eagerness, yielding an increase of MVPA of ~5–6 min per day. Our findings thus suggest that EPA has the potential to predict physical activity levels in adolescents, which supports the conclusion of an earlier study by Säfvenbom et al. (16) of eagerness and physical activity among young adults. This association may also be interpreted as indicating that eagerness may express the interaction between behavioral, cognitive and affective aspects, thus representing qualities which do not only predict physical activity, but also meaningful and personally relevant physical activity. According to previous research (68–70), which identifies the importance of highlighting and exploring positive emotions and experiences of meaning in order to understand adolescents' physical activity participation, the results of this study can be considered as a relevant scientific contribution to the literature.

It is, however, important to point out that the association between EPA and MVPA is no more than moderate. According to Nasuti and Rhodes (70), this may be related to how physical activity levels are objectively measured, thus capturing spontaneous activity, which is not included in the assessment by young people's relationship to physical activity in specific contexts. In Nasuti and Rhode's (70) study, affective assessments incidentally correlated better with self-reported physical activity. The association between EPA and MVPA in this study can thus be interpreted in light of the fact that the participants belong to 18 local schools, which are located close to their home environment. This makes it probable that many walk or bike to school, which is a type of physical activity registered by an accelerometer, but less so when assessing one's own relationship to physical activity. It may therefore be plausible that the association between eagerness and MVPA would have increased if eagerness had been analyzed according to intentional physical activity and more activity-specific contexts. This perspective can be further strengthened by the results from Basset et al.'s (71) review study, which demonstrated that among all the interventions in school and the built-up environment, active transport to and from school was one of the factors which contributed to the greatest increase of daily physical activity.

The findings also demonstrate that perceived parental support has a positive impact on the adolescents' EPA. This result bolsters prior research (27, 30, 72), which reports that the activities of parents, and their attention to, and support of, their children, influence how the youths experience and assess the importance of physical activity in their own lives. Although levels of perceived parental support generally decline with age, especially during early and middle adolescence (31), the participants in this study (age 12–13 years), still confirmed the importance of parents in relation to EPA.

Mean values for PAC reveal that, on average, the young people assessed their competence as medium high, and correlation analyses between PAC and EPA show that PAC had a moderately strong connection to EPA. This result may suggest an interpretation of the relationship between PAC and EPA as circular.

The SEM analysis shows that PAC, in addition to EPA, possesses significant explanatory power related to MVPA. This result confirms the positive association between feeling competent and involvement in physical activity, as reported in previous work (12, 73–75). When the path between eagerness and MVPA is mediated by PAC (Figure 1), the explanatory power of MVPA increases, without any change in the direct path between eagerness and MVPA. This means that the relationship between eagerness and MVPA is not conditional on PAC, but that the explanation of variation of MVPA is strengthened when the importance of both EPA and PAC is considered.

It is also worth noting that the total model can only explain 17% of the MPVA variation. In addition to what has been previously discussed, a further explanation could be linked to Weiss and Phillips (17), who points to cognitive maturity level and social-environmental dimensions, such as social comparison and evaluation, as key contributors to reflection on, and self-appraisal of, ability. Based on the age of the cohort, and the social conditions and reference systems in school and sports, which constitute decisive premises for experiencing and assessing competence and performance (69), it is possible that PAC is less able to predict physical activity for this age group than what has been proven for older youths.

However, given the fact that there is a weak, but still significant association between the study correlates and the participants' level of MVPA, we consider it to be important that all persons, contributing as resources in the youth's local community, aim to amplify the young people's eagerness for physical activity. This can be a process within the family, with parents or siblings who can communicate a positive value-orientation toward physical activity (25), act supportively toward their off-springs physical activity behavior, and serve as physically active role-models (29). In physical education, and perhaps even more so in sports, we point to Schenker (76) and Haugen (19), and put forward the idea of placing the young person at the center of attention, instead of the activity or sport. A great diversity of adolescents attend sports clubs or PE, and to be able to promote physical activity as a personally relevant, delightful and attainable experience, we consider the intention of seeing the individuals prerequisites, needs and desires to be of significant importance.

### Strengths and Limitations

A strength of this study is that objective and validated measurements of physical activity have been administered with 302 youths. However, as this is a cross-sectional study, the risk of non-response bias (77) must be considered when interpreting the study results. Another limitation in a cross-sectional study like this is that it does not allow for causal interference (11). Our measurement model is therefore based upon theoretical constructions and previous research. Other measurement models could certainly have been explored, but in this study our main issue was to pursue how EPA impacted on MVPA. Furthermore, we intended to examine how adolescents' perception of parental physical activity attitude and support, relates to EPA. Acknowledging also that PAC potentially could influence EPA, the alternative model was tested. In this sample, this alternative model didn't yield acceptable model-fit, but in a longitudinal study, this would be an interesting issue to explore. Measurements of MVPA with an accelerometer have been utilized in previous studies, and constitute a recognized and standardized way of measuring the physical activities of young people (1, 8). Nevertheless, a weakness in this measurement equipment is that activities with horizontal movements (e.g., cycling) or activities in water (e.g., swimming) are not measured. Data from the questionnaire revealed however that 83.3% of the participants had not been to the swimming pool during the week they wore the accelerometer, 8.8% had been to the swimming pool once, and 7.9% had been to the swimming pool twice or more. Inclusion of the participants water activity would probably have increased the average MVPA level, but nevertheless not large enough to affect the interpretation of overall physical activity measurement in the study. The questions formulated in the Eagerness for Physical Activity Scale (EPAS) have previously been tested and validated, but lack validation in cohorts younger than 15 years of age. The relevance of sociocultural factors on variance in EPA, was explored through perceived parental support. According to previous research (78), other sociocultural aspects, such as socioeconomy could also have been applied in our study.

## Conclusion

The findings show that youths who have a higher degree of eagerness for physical activity tend to be more physically active (MVPA), and accordingly better pre-requisitions for gaining good health. Furthermore, perceived athletic competence adds an indirect effect to MVPA, although without a decline in the effect of eagerness on MVPA. In addition, parental support exhibited a significant positive association with their children's eagerness. Even though the interaction of factors that might explain the physical activity level of adolescents comprises more factors than illuminated in this study, the importance of promoting the experience of delight, and personal relevance in physical activity, nurturing hopes, and intentions to maintain physical activity in the future (i.e., EPA) are confirmed as significantly important among 12–13-years-old in this study. Studies on change in this association with age should be further explored. Bearing in mind that this study's total model could only explain 17% of the MPVA variation, future research should also continue to explore other personal and/or environmental correlates of adolescents physical activity behavior.

## Ethics Statement

The subjects were fully informed about the protocol prior to participating in the study. A written consent form was signed by the parents of the adolescents, according to accepted ethical research regulations. Approval to use the data and conduct the study was given by the Norwegian Social Science Services (NSD).

## Author Contributions

HM, PL, and RS contributed to the design and methods and the writing of the introduction, methods, results, discussion, and conclusions. MB contributed to the design and methods and writing of methods. HM, PL, MB, and RS contributed to the critical review of the article.

### Conflict of Interest Statement

The authors declare that the research was conducted in the absence of any commercial or financial relationships that could be construed as a potential conflict of interest.
